# Men’s perception of information and descriptions of emotional strain in the diagnostic phase of prostate cancer—a qualitative individual interview study

**DOI:** 10.1080/02813432.2021.2004734

**Published:** 2021-11-21

**Authors:** Maja Elisabeth Juul Søndergaard, Kirsten Lode, Svein Reidar Kjosavik, Sissel Eikeland Husebø

**Affiliations:** a Department of Surgery, Stavanger University Hospital, Stavanger, Norway; b Research Group of Nursing and Healthcare Sciences, Stavanger University Hospital, Stavanger, Norway; c The General Practice and Care Coordination Research Group, Stavanger University Hospital, Stavanger, Norway; d Faculty of Health Sciences, University of Stavanger, Stavanger, Norway

**Keywords:** Diagnostic phase, emotions, information, patient experience, prostate cancer, qualitative research

## Abstract

**Objective:**

To explore men`s perception of information and their possible emotional strain in the diagnostic phase of prostate cancer.

**Design, setting, patients:**

A qualitative explorative research design was employed. Data were collected from June to November 2017. The study was set at a urological outpatient clinic at a university hospital in Norway. Semi-structured interviews were conducted with ten men who had been examined for prostate cancer. Interviews were analyzed using Systematic Text Condensation (STC).

**Results:**

The analysis revealed three themes. The theme ‘Different needs and perceptions of information’ illustrated that information should be personalized. Despite different information needs, insufficient information about prostate cancer may prevent some men from being involved in decisions. The theme, ‘A discovery of not being alone’, indicated that a sense of affinity occurs when men realize the commonality of prostate cancer. Some men benefited from other men’s experiences and knowledge about prostate cancer. The last theme ‘Worries about cancer and mortality’ showed that the emotional strain was affected by men’s knowledge of cancer and the received information. Men expressed conflicting feelings toward prostate cancer that could be difficult to express.

**Conclusions:**

The findings indicate that men in the diagnostic phase of prostate cancer are not a homogeneous group, but need personalized information. Some men may benefit from other men’s experiences and support. Men’s emotional strain can affect their communication about prostate cancer, which should be acknowledged. Procedures that identify patients’ information needs early on should be an integrated part of the diagnostic phase of prostate cancer.

**KEY POINTS**
- Knowledge about men’s information needs and possible emotional strain in the diagnostic phase of prostate cancer are limited.
- Men with suspected prostate cancer have different preferences and information needs; however, insufficient information prevents men from participating in decisions.
- Men experience a sense of affinity with other men affected by prostate cancer, and some men benefit from exchanging experiences.
- Men consider prostate cancer as a less aggressive type of cancer but may experience emotional strain.

## Introduction

Prostate Cancer (PCa) is the second most common cancer, with 2.3 million new cases worldwide in 2018 [1]. Prostate cancer is usually suspected based on prostate-specific antigen (PSA) levels and/or a digital rectal exam, but a definitive diagnosis depends on a biopsy that verifies adenocarcinoma in the prostate gland [2,3]. The European Assosiation of Urology (EAU) PCa Guidelines recommend that men are not subjected to PSA testing without being well-informed on potential risks and benefits [3].

Norway is among the countries with the highest incidence and mortality of PCa, with about 5000 annual incidents [4,5]. In Norway, general practitioners (GPs) perform an important function as a gatekeeper in selecting men for further diagnostic evaluation. There is consensus that a decision to have a PSA test should be made by patients in consultation with their GP who can explain the risks and benefits of the test, as well as risk factors and symptoms of PCa [6,7]. According to the Norwegian Directorate of Health, a large number of men without symptoms undergo PSA testing. They state that more information about the advantages and disadvantages of PSA testing will provide a better foundation for men’s decisions about the test [8].

Lack of information about cancer treatment and its adverse effects has been described as a common experience for patients during the process of PCa care [9]. In addition, studies have reported that men with PCa requested more timely and accurate information. Information on treatment and side-effects provided simultaneously with the diagnosis was considered too late [10,11]. Altered masculinity and sexuality, urinary incontinence and bowel changes have been identified as some of the challenges that men are confronted with after treatment [9,12,13]. A meta-synthesis of qualitative studies found that treatment of PCa represented a threat to masculinity due to a changed body and the challenged sense of being a man [14]. Evidently, information and emotional challenges are important focal points related to the trajectory of PCa.

Whilst several studies address men’s information needs and emotional challenges after a PCa diagnosis, few studies have focused on these aspects before a PCa diagnosis [15,16]. Little is known regarding men’s perception of the possible emotional strain in the diagnostic phase. Both Appleton et al. [17] and Biddle et al. [18] found that men experienced uncertainty and sometimes anxiety throughout the diagnostic pathway and information was identified as central to help manage these emotions [17,18]. Research shows that men are generally aware that PCa can pose an important health issue [19]. However, a systematic review of qualitative research found that the understanding of causes, symptoms, anatomy, and diagnosis of PCa is limited among undiagnosed men [15]. Studies have reported that men experience uncertainty when confronted with an elevated PSA level and future management plan [18,20].

A qualitative approach may contribute important knowledge about the diagnostic phase of PCa from a patient perspective. Therefore, this study aims to explore men`s perception of information, and describe the possible emotional strain in the diagnostic phase from an elevated PSA test result was recognized and until they received the biopsy result.

## Materials and methods

A qualitative, explorative descriptive design was employed [21]. This study adhered to the Standards for Reporting Qualitative Research checklist [22]. Semi-structured individual interviews were used as a data collecting method [23]. An interview guide was developed based on the aim of the study, the diagnostic pathway of PCa [6], and previous research. Interview data were analyzed using Systematic Text Condensation (STC) [24] to facilitate the descriptive and explorative approach, presenting participants’ experiences as expressed by the participants themselves [25].

### Setting and participants

The study was conducted at a urological outpatient clinic staffed by urologists and nurses at a university hospital located in an urban setting in the southwestern part of Norway. The interviews took place in consultation rooms located at the hospital close to the urological outpatient clinic, or a satellite clinic within the same health trust; others were held in conference rooms at the hospital. The first author and participants decided upon the interview locations together based on convenience for the participants. For example, some men wished to combine the interview with other appointments at the hospital. The hospital environment may have affected the participants’ descriptions of their experiences [23]. A purposive sampling strategy was used, which included variation in age and PSA level at the time of referral [21]. Eligible participants received the invitation to participate together with the study information from the first author while they awaited a prostate biopsy at the urological outpatient clinic. Participants who considered participating received further information about the interview in a consultation room. The following inclusion criteria were used: men scheduled for a prostate biopsy, first episode of PCa, ≥40 years of age, and able to provide informed consent. Men with cognitive impairment were excluded. Participants were re-contacted by the first author after they received their biopsy result, i.e. ∼2 weeks after the biopsy. None of the participants chose to withdraw from the study.

### Data collection

Data were collected between June 2017 and November 2017. Demographics were obtained from the participants before the interviews. All interviews were audiotaped. To begin each interview, the participants were asked to tell about their experience from the time of their PSA test and during the diagnostic phase of PCa. Some participants had taken several PSA tests and they also spoke about the information they had received at the time of their first PSA test. The first author asked elaborating questions based on the participant’s own story. Further, the interview guide was used to ensure that common themes were reflected upon according to the aim of the study (see Supplementary Appendix 1). This included their experience with the PSA test, received information, their experiences with information about the medical procedures at the urological outpatient clinic, and their thoughts, emotions, and possible worries. The interviews lasted between 25 and 52 minutes. After ten interviews, the research team agreed not to perform any further interviews based on the study aim, sample specificity, the quality of the dialogues, and our analysis strategy [26].

### Data analysis

The recorded interviews were transcribed verbatim and analyzed thematically using STC [24]. The method consists of four steps: (1) total impression, from chaos to themes; (2) identifying and sorting meaning units, from themes to codes; (3) condensation, from code to meaning; and (4) synthesizing, from condensation to descriptions and concepts [24,25]. The first step was performed by the first author and, after gaining an overall impression, six preliminary themes were identified. In the second step, each transcript was systematically reviewed by the first author, and meaning units were sorted and classified into code groups related to the preliminary themes. The full transcripts were distributed within the research team and the preliminary analysis was discussed among the authors at an analysis seminar. The preliminary themes and codes were adjusted to ensure that they reflected the narratives and the aims of the study. Analysis benefits from being conducted by more than one researcher to create a wider analytic space. The first author performed the third step, which involved the abstraction of meaning. The content of each theme was reduced into a condensate reflecting the meaning of the text [25]. The research team then reduced and renamed the preliminary themes into three final themes. The fourth step required the condensates to be synthesized into generalized descriptions by the first author. The descriptions were then evaluated by the research team, ensuring that they reflected the original context.

This research team consisted of an experienced GP, two researchers in nursing with experience in conducting qualitative studies, and one specialist nurse, all of whom had previous experience with patients affected by cancer. The first author is a Ph.D. fellow and a former cancer coordinator for bladder and kidney cancer but also served as a PCa coordinator on various occasions. This position was located at the hospital and involved some acquaintance with the urological outpatient clinic. The multidisciplinary composition of the research team strengthened the process of reflexivity during the analysis. Selected parts of the analysis process are shown in Table 1.

**Table 1.** Examples of the analysis (selected parts).

Meaning units (selected)	Codes	Subthemes	Themes
I knew that he had taken a blood sample, but I did not know that he had examined the PSA and neither had he done a finger exam of the prostate gland. (ID40 p2)	Different information and patient involvement throughout the diagnostic phase		Different needs and perceptions of information
He [GP] was saying that there [at the hospital] they go in and examine the prostate, but he did not say anything more specific as far as I can recall, so after this first consultation or a couple of consultations, I had no clear idea of what follows. (ID5 p3)	Different information and patient involvement throughout the diagnostic phase		Different needs and perceptions of information
I think to myself that I am not the first person to go through this, and not the last either. (ID38 p3)	Different experiences with social support	One of many	A discovery of not being alone
As a human being, you often imagine the worst possible outcome, it is easier to think the worse than the best. You hope for the best but prepare for the worst… It's a human weakness, I guess. (ID40 p3)	Different experiences and psychological reactions	Feelings of uncertainty toward cancer	Worries about cancer and mortality

### Ethical considerations

The study was approved by the Norwegian Regional Committees for Medical and Health Research Ethics (REK no. 2017/71) and the hospital administration. The participants received both oral and written information about the study and were guaranteed anonymity. Participation was voluntary and participants were informed that they had the right to withdraw from the project at any time. All participants signed a letter of consent before the individual interview started.

## Results

**Figure 1.**
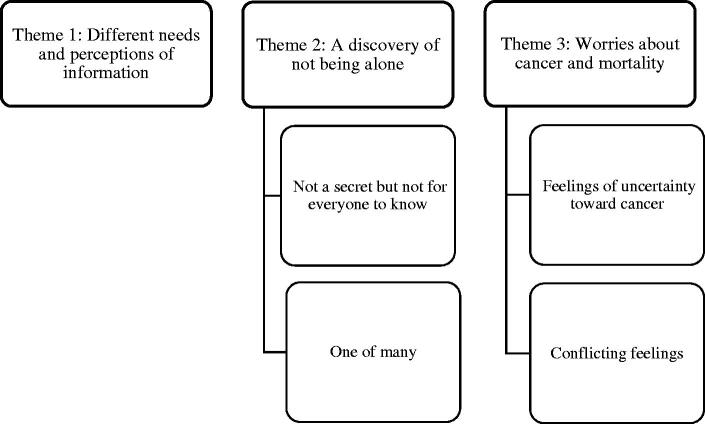
Main themes with associated subthemes.

Ten men ranging from 50 to 80 years old participated in the study; six of them were diagnosed with PCa, while the remaining four participants had benign biopsies (Table 2). Participants with benign biopsies received no PCa diagnosis but had to continue to monitor their PSA level regularly with their GP. They expressed satisfaction with being monitored for safety reasons. Three themes with related subthemes emerged from the analysis (Figure 1): (1) Different needs and perceptions of information, (2) A discovery of not being alone, and (3) Worries about cancer and mortality.

**Table 2.** Sample demographics.

Variables	*n* = 10
Age (years)	
51–60	1
61–70	6
71–80	3
People in the household (*n*)	
1 person	3
2 persons	6
≥3 persons	1
Education, after primary school (years)	
1–3	1
4–5	6
7–9	3
Occupation status (*n*)	
Employed	4
Retired	5
On rehabilitation	1
Prostate cancer (*n*)	
Yes	6
No	4

### Theme 1: different needs and perceptions of information

The first theme addressed the participants’ perception of the received information from healthcare providers during the referral process and consultations at the outpatient clinic. The theme reveals that men have different needs for information and involvement in decisions. Being unaware that the GP had ordered a PSA test was a common experience among the participants. They described that their GP ordered the blood sample without giving further information about the actual reason for the test. A participant explained that he had consulted the GP because of pain in the groin area. The GP suggested that the pain could be caused by an enlarged prostate gland. After a pause of thinking, the participant explained that he was unsure if the GP had mentioned the word ‘cancer’. The participant had not received any information regarding the PSA test; therefore, he had not understood the relationship between the blood test and PCa.

*Just now I was thinking what the reason might be … I have not felt that he did it behind my back, since he is a really nice and open-hearted person and I don't think he tried to hide anything from me. So why this value [PSA] was measured, whether it was by chance or not, I don't know. (ID5 p2)*

Whether the GP ordered the PSA test with or without the participants’ knowledge, the participants perceived the GP’s decision as a result of his/her competence and care. Participants were unprepared when receiving the PSA results without being aware of the PSA test in the first place. A participant described how his GP had called him and explained about the PSA test and the possibility of PCa:

*He [my GP] said it indicates that it may be cancer that was what he said, so I … but he also said it can be an indication that you have an infection. I didn't know that PSA was to indicate prostate cancer, I had no idea. (ID30 p4).*

The cancer had not been discussed before the blood test so he thought the blood test was only related to an infection. He expressed a concern that healthcare providers assumed that ordinary people were familiar with medical terms and procedures, which he believed was a general problem during the diagnostic phase of PCa.

Insufficient knowledge and information seemed to produce uncertainty and reduce predictability, and thereby possibly diminishing participants’ will and opportunity to be involved in decisions about their own health. However, some participants stated that they preferred to refrain from being included in medical decisions and avoid unnecessary information.

*I was happy with the information given in writing. I don't have, well, I am a bit like I take things as they come, I don't need to know absolutely all there is to know about everything. (ID38 p5).*

These participants seemed certain that the healthcare providers possessed the necessary competence and should be allowed to perform their job without them interfering or questioning their actions. Information provided together with the results of the elevated PSA test or the prostate biopsy may have caused this attitude. Participants did not feel prepared for a co-responsibility, because the decision itself could lead to pondering and feelings of uncertainty and guilt.

The results demonstrated variation related to the biopsy-based on their perception of the information, their expectations, and subsequent sequelae. A participant explained that the healthcare providers were friendly and caring, but failed to provide predictability:

*Absolutely nothing [of information] has come through the system. No, the biopsy… when I came in for a biopsy, I came in and they took a sample, and that was that [The participant’s prior perception of a prostate biopsy], but then time went on and on and on, and I thought, heavens! – By now they must be finished! Because no-one had told me what it was actually all about; sure, I could perhaps have found out for myself, but I think the information was not as good as I had expected. (ID30 p3)*

Participants who requested more information felt poorly prepared both before and after the biopsy. They expected information to prevent them from wondering about normal bodily reactions. Consequently, they searched the Internet for information, which should have been communicated by the healthcare providers. For instance, insufficient information regarding unsuspected prolonged bleeding after the biopsy seemed to arouse unpleasant feelings of worry and disgust.

### Theme 2: a discovery of not being alone

The second theme, ‘A discovery of not being alone’, consisted of two subthemes: ‘Not a secret but not for everyone to know’ and ‘One of many’. This theme highlights how relations could be an important source of support and information, especially men with similar experiences who were valued. The commonality of PCa created a sense of affinity for other men affected by PCa.

#### Not a secret but not for everyone to know

Participants were selective in whom they confided concerning the suspicion of PCa. The decision to inform the nearest family, friends, and coworkers often depended on a wish for support and honesty. Some participants described these conversations as beneficial due to increased emotional well-being when they shared their personal concerns and received support. A participant who lived alone exemplified this:

*In the course of time, I have spoken to 2–3 of my closest friends, and I see it is good for me, really it is. It is not something I blurt out to all and sundry, but I talk to my nearest and dearest. So, I am not anxious about that actually… no. (ID38 p2)*

For participants who needed more information and support, family members or friends with previous PCa experience contributed to a more understandable and participatory picture of the diagnostic phase. A participant explained:

*I have been fortunate; in the first place I have a buddy who is slightly ahead of me, and who has told me a lot, and that has also meant that I can go online [the Internet] and read for myself, but not everyone has that option. (ID43 p7)*

In contrast, participants exposed to what they explained as emotional overreactions or scary stories, tended to avoid discussing the subject with others, allegedly because it interrupted their perception of their current situation. Being reluctant to share the suspicion or diagnosis of PCa was apparently also based on an intention not to be a burden. In general, participants expressed that conversations with friends about PCa were related to practical issues because they rarely discussed feelings with each other nor felt the need. Conversations about feelings seemed to be reserved for their significant other.

#### One of many

Participants expressed a feeling of not being alone when they realized the commonality of PCa among their acquaintances. The acknowledgment of being one of many appeared to generate a feeling of affinity, which was unaffected by whether the participants had discussed the topic with others or not. The Internet and the media also contributed to this feeling, as a participant said:

*But then I also read what is online, and almost 40 percent of those over 75 have had problems with their prostate, so I am one of many. But then, most of them have a good outcome. (ID38 p3)*

This insight of being one of many may represent a significant contribution to how participants perceived themselves during the diagnostic phase of PCa as well as after a confirmed diagnosis. The realization seemed to direct their feelings toward hope and acceptance of the situation.

### Theme 3: worries about cancer and mortality

The third theme, ‘Worries about cancer and mortality’, consisted of the two subthemes: ‘Feelings of uncertainty toward cancer’ and ‘Conflicting feelings’. In this theme, participants expressed their emotional strain related to the suspicion of PCa. The information they received about PCa seemed to affect their emotions toward PCa.

#### Feelings of uncertainty toward cancer

The period leading up to a possible PCa diagnosis could be perceived as demanding, and uncertainty about the disease and the future was difficult to reconcile with. Participants’ prior knowledge and the information they received appeared to influence their emotional response to the suspicion of PCa. A participant described himself as anxious by nature, though the information and conversations with the healthcare providers had convinced him that there was little suspicion of PCa. However, before the first consultation at the outpatient clinic, he felt paralyzed with fear.

*When it comes down to it, it is the not-knowing and the uncertainty that can be the most tiresome, more than getting a clear message that you have cancer, this is until death. (ID5 p8)*

Participants spontaneously associated cancer with death, as they knew someone who had died from cancer. On second thought, however, the participants expressed beliefs that modern medicine provided many treatment opportunities that could possibly cure cancer. Prostate cancer was considered a less aggressive cancer illness but was nevertheless a reminder that life was fragile. Physical discomfort, such as abdominal pain and tension, difficulty sleeping, and catastrophic thoughts were described as reactions related to the suspicion of PCa. For some, concerns and stress became more prominent as the results of the biopsy approached. Participants assessed both the meaning and the impact of a potential PCa diagnosis:

*I did not worry over the MRI [magnetic resonance imaging] scan, but when I heard that there was something there, then your stress level goes up, and when you get closer to the biopsy, then it goes up even further, and when you are anxiously waiting for the answer, then I was at the peak emotionally. At that point you have come a whole step closer to somewhere you would rather not be. (ID43 p7)*

This was described as a process of sorting out one’s thoughts and included reflections on life and death, but also finding acceptance.

#### Conflicting feelings

It was common that participants appeared to experience conflicting feelings when describing their thoughts about PCa. This was expressed in reluctant and ambiguous statements during the interviews. For example, a participant stated that he had not spent time speculating; however, during the interview, he expressed anxiety about the illness and tried to avoid thinking about it:

*No, I did not think about it at all [PCa]. Well, yes, at the time I am a bit like, I think a little bit, well, I think a little bit like, if I think scary thoughts, then my thoughts tend to focus on cancer, you know, in a way that, well that's how it is, you know. (ID36 p3)*

After receiving a negative biopsy result, some participants described themselves as feeling surprisingly relieved and more energetic.

## Discussion

The findings demonstrate that men in the diagnostic phase of PCa are not a homogeneous group but have different needs for information. Men experienced a feeling of affinity when realizing the commonality of PCa. The received information and their prior knowledge about PCa seemed to affect men’s emotional response to the suspicion of PCa. However, the suspicion of PCa often evoked a sudden fear of death but was nevertheless perceived as a less dangerous type of cancer.

In general, participants in this study experienced information regarding the PSA test as inadequate or untimely to provide them a framework within which to act. These findings are in line with previous studies on shared decision-making in PSA screening, which found that the decision on the PSA test was not often patient-centered [27,28]. This clinical practice diverges from both Norwegian and European guidelines, which strongly recommend providing thorough information before a PSA test for men to participate in the decision-making [3,8]. However, the results of the current study emphasize variations in men’s preferences for healthcare providers to make decisions. Participants expressed general confidence in their GPs, although the discussion about possible benefits and harm seemed insufficient. A recent study argues that shared decision-making during a diagnostic phase of an illness is very complex and should be considered from the perspective of the patient and family. The results emphasize the importance of context and acknowledge that although doctors are the most trusted source of health information, patients may obtain information from various sources. The decision-making is thereby expanded beyond the boundaries of the medical visit [29]. A previous study found that GPs’ communication practice in relation to PSA testing vary, specifically about overdiagnosis of PCa. Difficulty in understanding the concept, being contradictory to existing health beliefs and the perception of cancer as dangerous has been identified as contextualizing factors that prevent communication [30].

Most participants in the present study expressed genuine confidence and humility towards the healthcare system. Although participants expressed that information about the diagnostic process and what to expect was important, some preferred the healthcare providers to make the decisions and keep the information at a basic level. Previous studies have found that not all patients desire to be involved in decisions about their treatment after being diagnosed with PCa [31,32]. Findings in the current study suggest that men will benefit from their individual information preferences and wishes for involvement being identified early in the diagnostic phase of PCa. In Norway, patients have a legal right to receive information to gain insight into their own state of health and treatment, as well as possible side effects. However, information should not be given contrary to the patient's own request unless an omission causes harm [33]. Patients may have different preferences for being involved in decisions but this preference should be informed rather than based on clinicians’ presumptions [34]. Therefore, personalized information should be available for patients in the diagnostic phase of PCa.

Although some participants in our study were reluctant to tell others about the suspicion of PCa, most participants told their closest family and friends. Similarly, a review study found that men often place restrictions on the circle of people who know about the suspicion of PCa. The restraint was described as an attempt to maintain masculinity and protect themselves from any potential stigma. This behavior has been suggested as a final line of resistance against the dissemination of knowledge about PCa [35]. In contrast, most participants in the present study valued conversations and exchanges of knowledge with other men with similar experiences. This is in line with previous research on men’s behavior after a PCa diagnosis, which showed that men with PCa seek different types of information. In addition to medical information, many men valued lived-experience information that came from sources with direct and personal connections to the information. This contributed to sympathy from others and helped to understand the different types of treatment [32]. Furthermore, previous research has revealed commonality as an important factor for whether men disclosed their PCa diagnosis. By recognizing others with similar experiences, men’s desire to share their diagnoses and exchange knowledge was stimulated [36]. Our findings suggest that men in the diagnostic phase of PCa can combine information from healthcare providers with the experiences of others to understand their own situation and the possible consequences. Nevertheless, issues affecting emotions related to PCa were rarely discussed and often reserved for their significant other. Regardless of how much the participants chose to share about the suspicion of PCa, the commonality of men affected by PCa was experienced as comforting and contributed to a sense of affinity.

The findings in this study showed that the participants managed the suspicion of PCa in a continuum, from no worries to thoughts of death. Despite general anxiety about cancer, PCa was considered a less aggressive cancer. This assumption was often reinforced by other men’s experiences or from information on the Internet. Some participants seemed to have conflicting feelings towards their own experiences and thoughts about PCa, which resulted in reluctant or ambiguous statements. According to a recent review, the construct of masculinity is a major barrier for men to seek prostate care because of a perceived prohibition of displaying weakness. The fear of being perceived as weak was found to limit health information seeking and communication about prostate issues. Existential fear could also be perceived as a sign of weakness. Eventually, this construct of masculinity made some men avoid situations that could make them appear weak [15]. This further emphasizes the importance of an approach that maps individual beliefs and experiences that affects their health behaviors [37].

### Strengths and weaknesses of the study

Since men in the diagnostic phase of PCa are in the transition between primary- and specialist healthcare, this study may be valuable to healthcare providers at both levels. A qualitative method was used to meet the study’s aim and to explore an area with limited knowledge. The research team contributed with different perspectives and experiences, which strengthened the analysis. The first author's previous close relation to the affected patients made it easier to elaborate on certain parts of the interview and obtain deeper descriptions. Nevertheless, this relation could also represent weakness, and therefore, uncovering preconceptions was highly prioritized to ensure objectivity and that the data represented the information given by the participants. In addition, we have presented a rich description of the participants, the setting, and the analysis to promote transferability. To establish confirmability, we have described the analytical process in which all the authors participated.

Due to the relatively broad aim, the number of participants who were recruited from one hospital is considered a limitation of the study. However, the analysis revealed a significant mutual influence between the participants’ information needs and their emotional strain. This finding has been little described in previous studies and thus the research team decided it may be of importance to clinical encounters. Although our results are related to the specific context and cannot be generalized, they may serve as a base for further research. Six of the ten participants were between 61 and 70 years, which gave a slightly uneven distribution of participants according to the intended sampling strategy of a wide variation in age. However, due to the fact that the median age of men diagnosed with PCa in Norway is 70 [5], it was agreed to be acceptable. Additionally, the first author recruited all participants, which may have resulted in sample bias [24]. Directly applying to patients could have made it difficult to say no.

## Conclusion and implications for research and practice

The findings in this qualitative individual interview study of men’s perception of information, and their emotional strain in the diagnostic phase of PCa emphasize the need for a flexible and tailored information structure that meets individual requirements. By offering patients information that is adjusted to their individual preferences, healthcare providers might reduce uncertainty and worry in the diagnostic phase of PCa. More research on how to address or screen patients’ information needs is required. The findings elaborate that some men value exchanging practical knowledge with other men with similar experiences. These experiences helped participants navigate the diagnostic phase and enhanced the feeling of being ‘one of many’. The recognition of not being alone added a sense of affinity, which applied to all participants regardless of where they obtained this knowledge. Healthcare providers should address this subject and direct men to patient organizations or the like if needed.

This study also offers new insight into how men experience the diagnostic phase of PCa and highlights that men may experience emotional strain that can be considered difficult to communicate, which may affect the dialogue between patients and healthcare providers.

## Supplementary Material

Supplemental Material
